# Posttranslational modification of Argonautes and their role in small RNA-mediated gene regulation

**DOI:** 10.1186/1758-907X-2-5

**Published:** 2011-09-26

**Authors:** Michael Johnston, Gyorgy Hutvagner

**Affiliations:** 1 RNA Biology, Department of Biology, Swiss Federal Institute of Technology Zurich, LFW D18.1 Universitätstrasse 2, 8092, Zürich, Switzerland; 2 Centre for Health Technologies, University of Technology Sydney, Sydney, NSW 2007, Australia; 3 Wellcome Trust Centre for Gene Regulation and Expression, College of Life Sciences, University of Dundee, Dow Street, Dundee, DD1 5EH, UK

**Keywords:** posttranslational modifications, phosphorylation, ubiquitination, Argonautes, miRNA, RNAi

## Abstract

Shortly after their discovery, repertoires of miRNA were identified, together with proteins involved in their biogenesis and action. It is now obvious that miRNA-mediated gene regulation itself is regulated at multiple levels. Identifying the regulatory mechanisms that underpin small RNA homeostasis by modulation of their biogenesis and action has become a key issue, which can be partly resolved by identifying mediators of Argonautes turnover. An emerging theme in the control of Argonaute stability and activity is through posttranslational modifications, which are the focus of this review.

## Introduction

Small RNA such as miRNA and siRNA have emerged as important eukaryotic posttranscriptional gene regulators. Functioning as guides, these small RNA direct Argonaute proteins to complementary targeted mRNA, often resulting in reduced gene expression by a variety of mechanisms [1-3]. Since the discovery of small RNAs, much focus has been directed toward dissecting their mode of action. Very recently, an increasing number of studies have also started to reveal mechanisms for the turnover of miRNAs [4,5]. However, the mechanisms that mediate the stability and activity of the pathways' central protein components, Argonautes, are less understood and are the topic of this review. We summarise studies that have identified posttranslational modifications of Argonaute and Piwi proteins and how these modifications affect Argonautes' function and/or turnover. We also reflect on the potential wider biological implications of these posttranslational modifications on gene silencing by modulating Argonautes' activity and/or turnover.

### Posttranslational control of Argonautes

Studies of a variety of organisms have shown that deletion or overexpression of the enzymes involved in miRNA biogenesis and action can severely disrupt major cellular process. Therefore, it is a cellular necessity to maintain the homeostasis of these components, which can be dynamically regulated in response to internal or external stimuli. An elegant example of this is a negative feedback loop that exists in Arabidopsis. Where AGO1, the main Argonaute directing miRNA and siRNA silencing in plants, is involved in a posttranscriptional autoregulatory loop with a low-abundance miRNA, miR168 [6]. Only when AGO1 protein levels increase is miR168 able to incorporate into the RNA-induced silencing complex (RISC) efficiently and target *AGO1 *mRNA. The inverse is also true. When AGO1 protein levels decrease, miR168 is less effectively loaded into RISC and a consequent *AGO1 *mRNA increase is observed; thus small perturbations are compensated for to maintain *AGO1 *equilibrium [7]. An *ago1 *mutant resistant to miR168 silencing shows increased mRNA expression but developmental defects [8]. To date, analogous regulatory mechanisms for mammalian Argonautes have remained elusive; however, a few recent findings have indicated that these Argonautes undergo a barrage of signals that mediate their action and stability. A recent study has demonstrated that Ago2 is the most abundant human family member at both the mRNA and protein levels in a wide range of commonly used cell lines [9]. In contrast, Ago4 protein levels are barely detectable, even though relatively high levels of mRNA have been detected, suggesting that, at least for Ago4, posttranscriptional control may in part mediate its expression [10,11]. Similarly, Argonaute proteins have also been found to harbour a variety of posttranslational modifications, each with differing degrees of conservation between the four human isoforms (Additional file 1, Table S1). Posttranslational modifications manifest as chemical modifications that occur on amino acid side chains in a site-specific manner. They can temporarily or permanently change the fate of the protein by enhancing the functionality and/or stability of the target protein through the recruitment of auxiliary factors, change the proteins' cellular localisation or signal the most terminal fate, proteasomal degradation.

Qi and colleagues [12] demonstrated that human Argonautes can undergo prolyl 4-hydroxylation by type I collagen prolyl 4-hydroxylase (C-P4H9I). They showed *in vitro *that Ago2 and Ago4 were more susceptible to hydroxylation than Ago1 and Ago3. In the case of Ago2, prolyl 4-hydroxylation of proline 700 (P700) is necessary for stability. Depletion of a C-P4HPI subunit, P4H-α, leads to a decrease in Ago2 protein level and subsequent reduction in RNAi efficiency. However, prolyl 4-hydroxylation appears to be dispensable for miRNA-mediated translation repression. Furthermore, the Ago2 P700A mutant, containing a mutation that prevents prolyl 4-hydroxylation, could partially rescue RNAi if endogenous Ago2 is depleted [12]. Although it remains to be determined whether hydroxylation enhances small RNA binding, the decreased stability observed with the Ago2 P700A mutant is perhaps, a consequence of this.

A more recent study has linked poly(ADP-ribose), or pADPr, to modification of Argonautes by a subset of pADPr polymerases. In the presence of stress, all four human Argonautes seem to be modified, with overall relief of miRNA-mediated silencing, being observed [13].

A few studies have associated phosphorylation with an ability to regulate Argonautes' function and localisation. For example, oxidative stress directed by sodium arsenite induces phosphorylation of serine 387 on Ago2 through p38 mitogen-activated protein kinase. Interestingly, this site is conserved in human Ago1 and Ago4, but not in Ago3 [14]. An increase in Ago phosphorylation may in part explain the general increase in global miRNA expression observed previously with sodium arsenite treatment [15]. Phosphorylation also facilitates Argonautes' P-body localisation [14]. Researchers at the Meister laboratory [16] recently discovered that Ago2 can be phosphorylated on seven different amino acid side chains, many of which coincide within known functional domains. Their main efforts focused upon tyrosine Y529, which is located in the MID domain and conserved in a wide range of species. A combination of structural studies and the creation of a constitutive phosphorylation mimic by the substitution of Y529 with negatively charged glutamate revealed that phosphorylation can inhibit small RNA binding by creating a negatively charged environment within the small RNA 5' end binding pocket, thus opposing the 5' phosphate of the small RNA. This therefore could provide an elegant switch mechanism by which to regulate unloading of Argonautes and prevent gene silencing. Furthermore, this would inherently mediate Argonautes' turnover, as Argonautes bound to small RNA are considerably more stable than the unloaded form [17].

The vast majority of intracellular proteins are degraded by the ubiquitin-proteasome system, and Argonautes are no exception. The proteasome is responsible for degrading damaged, misfolded and redundant proteins. Specific degradation is accomplished by the actions of ubiquitin, which is covalently bound to a lysine residue on the targeted substrate. *mLin41 *(mouse homologue of *lin-41*), which is targeted by *let-7 *specifically in stem cells, has been reported to act as an E3 ubiquitin ligase for Ago2. mLin41 directly binds and ubiquitinates Ago2, thus acting as a negative regulator of the miRNA pathway. Alterations in mLin41 levels inversely affect Ago2 stability by recruitment of the proteasome [18]. A potentially analogous mechanism may exist in plants, wherein the F-box protein FBW2, a modular component of the Cullin-RING E3 ubiquitin ligase, has been reported to be a negative regulator of Ago1 in *Arabidopsis thaliana *[19]. Other mammalian E3 ligases have also been implicated in a regulatory role for miRNA-mediated gene regulation. The TRIM-NHL family of proteins have been shown to influence miRNA-mediated gene repression [20-22]. Mammalian Trim32 contains a RING finger domain that confers E3 ligase activity. One of the Trim32 substrates was identified as the transcription factor c-Myc [21], which itself has been shown to downregulate miRNA expression at the transcriptional level [23]. Thus Trim32 has been shown to enhance the repression ability of certain miRNA indirectly but also directly by interacting with Ago1. However, Trim32 was not reported as being capable of ubiquitylating Ago1 [21]. Ubiquitination does not solely seal a protein's fate to the proteasome but also is involved in sorting proteins into multivesicular bodies and cell signalling networks. Therefore, one could postulate that tagging Argonautes at different stages with different ubiquitin chains could mediate their function and localisation. Initial studies demonstrated that human Ago2 can be associated with cell membranes [24], and more recent studies have linked Ago2 and GW182 to multivesicular bodies; therefore, it is not hard to imagine these trafficking event's being driven by ubiquitination [25].

Other posttranslational modifications have been linked to the turnover of the related Piwi proteins. *Drosophila *methyltransferase 5 (dPRMT5) catalyses the methylation of Ago3 and Aubergine, which enhances their stability. Additionally, methylation of Piwi proteins facilitates the recruitment of multiple members of the Tudor family, which may assist in piRNA production and loading of Piwi proteins [26]. Depletion of dPRMT5 contributes to a loss of piRNA and an accumulation of retrotransposons [27]. PRMT5 has also been shown to associate with human Argonautes [28], which is surprising, as motif-based predictions indicate the absence of any potential methylation sites in all four human Argonautes [27].

### miRNA homeostasis could be regulated via the regulation of Argonaute stability

The majority of miRNA research has focused on cataloguing changes in miRNA expression in diverse biological pathways and disease models. However, relatively little has been done to reveal the nature of the alteration of miRNA expression. In addition, we do not know how the endogenous miRNA pathway can deal with the sometimes immense influx of endogenous and viral miRNAs, which is characteristic of some immune cells upon infection [29]. As miRNAs have surfaced as key regulators for many different cellular and pathological processes, it is of little surprise to learn that they themselves are strictly regulated by a multitude of mechanisms. Mammalian miRNAs are regulated at the transcriptional level, with tissue and developmental stage specificity being key to their production. Another emerging theme conveys regulation at a posttranscriptional level. Many studies have identified additional components of the miRNA maturation pathway that alter the processing of certain pri- and/or pre-miRNAs. It is also evident that these auxiliary proteins can themselves respond to external stimuli and mediate the production of pre- and/or mature miRNA transcripts on demand, adding a further layer of regulation [30-33]. An additional stage at which miRNA homeostasis could be regulated is the point when miRNA are loaded into Argonautes. Previous studies have demonstrated that Argonaute expression can be the rate-limiting step for miRNA maturation. Overexpression of any of the four human Argonaute proteins leads to an increase in mature, ectopically expressed miRNA [34]. Lower miRNA expression has been observed in Ago2-knockout mice. However, overexpression of Ago2 in these cells could compensate and rescue miRNA levels [34]. Furthermore, overexpression of Ago2 has been found to enhance RNAi [35]. Similarly, we have previously demonstrated that the stability of unloaded Argonautes is different from that of Argonautes bound to a small RNA. Also, inhibition of heat shock protein 90 that stabilises small RNA-free Argonautes leads to the proteasome-mediated degradation of Argonautes. Ubiquitination, and potentially even SUMOylation, are indeed good candidates for the regulation of miRNA homeostasis by influencing Argonautes' stability and turnover. For instance, these pathways are frequently modified and hijacked by pathogens, which can also affect general miRNA homeostasis, either by introducing small RNA derived from the pathogen or by modulating components involved in miRNA biogenesis [36,37]. Moreover, misregulation of ubiquitination and SUMOylation could lead to uncontrolled cell proliferation and transformation, which are often characterized by alteration in the miRNA expression profile.

A series of systematic studies are necessary to identify and map all Argonaute posttranslational modifications in an effort to gain insight into the degree of posttranslational control. It is likely that these modifications act in concert to partition Ago in various cellular localisations, aid in the recruitment of auxiliary protein factors to build RISC and potentially determine when RISC is recycled. Furthermore, studies aimed at identifying how the miRNA pathways respond to attacks by pathogens may also lead indirectly to the identification of novel Argonaute posttranslational modifications and shed light on how miRNA homeostasis is maintained, all of which are necessary to gaining a full insight into the currently elusive mechanism behind Argonaute regulation and miRNA homeostasis.

## Abbreviations

miRNA: microRNA; pri-mRNA: primary microRNA; RNAi: RNA interference; siRNA: small interfering RNA.

## Competing interests

The authors declare that they have no competing interests.

## Authors' contributions

MJ and GH contributed equally to the writing and drafting of the manuscript. Both authors read and approved the final manuscript.

## Supplementary Material

Additional file 1 **Table S1. Posttranslational modification conservation of site-specific residues on Argonautes involved in miRNA-mediated gene regulation**. Tick represents residue that is fully conserved and predicted to be modified. Cross represents residue that is not conserved and therefore will not be modified. For posttranslational modifications where site-specific residue has yet to be determined, "Not tested" represents that there is insufficient evidence to suggest whether posttranslational modification is present, whereas tick indicates that the Argonaute has been experimentally shown to be modified. Click here for file
